# Characterization of Shiga Toxin 2a Encoding Bacteriophages Isolated From High-Virulent O145:H25 Shiga Toxin-Producing *Escherichia coli*

**DOI:** 10.3389/fmicb.2021.728116

**Published:** 2021-09-08

**Authors:** Silje N. Ramstad, Yngvild Wasteson, Bjørn-Arne Lindstedt, Arne M. Taxt, Jørgen V. Bjørnholt, Lin T. Brandal, Jon Bohlin

**Affiliations:** ^1^Department of Microbiology, Division of Laboratory Medicine, Oslo University Hospital, Oslo, Norway; ^2^Institute of Clinical Medicine, University of Oslo, Oslo, Norway; ^3^Department of Paraclinical Sciences, Norwegian University of Life Sciences, Oslo, Norway; ^4^Faculty of Chemistry, Biotechnology and Food Science, Norwegian University of Life Sciences, Ås, Norway; ^5^Department of Infectious Diseases and Prevention, Norwegian Institute of Public Health, Oslo, Norway; ^6^Division of Infection Control and Environmental Health, Norwegian Institute of Public Health, Oslo, Norway; ^7^ECDC Fellowship Programme, Public Health Microbiology Path (EUPHEM), European Centre for Disease Prevention and Control (ECDC), Solna, Sweden

**Keywords:** Shiga toxin-producing *E. coli*, whole genome sequencing, bacteriophage, Oxford Nanopore Technologies (ONT), Stx2a phage, virulence factors

## Abstract

Shiga toxin-producing *Escherichia coli* (STEC) may cause severe disease mainly due to the ability to produce Shiga toxins (Stx) encoded on bacteriophages. In Norway, more than 30% of the reported cases with STEC O145:H25 develop hemolytic uremic syndrome (HUS), and most cases, with known travel history, acquired the infection domestically. To describe phage characteristics associated with high virulence, we extracted the Stx2a phage sequences from eight clinical Norwegian O145:H25 STEC to conduct in-depth molecular characterization using long and short read sequencing. The Stx2a phages were annotated, characterized, and compared with previously published Stx2a phages isolated from STEC of different serotypes. The Norwegian O145:H25 Stx2a phages showed high sequence identity (>99%) with 100% coverage. The Stx2a phages were located at the integration site *yciD*, were approximately 45 kbp long, and harbored several virulence-associated genes, in addition to *stx2a*, such as *nanS* and *nleC*. We observed high sequence identity (>98%) and coverage (≥94%) between Norwegian O145:H25 Stx2a phages and publicly available Stx2a phages from O145:H25 and O145:H28 STEC, isolated from HUS cases in the USA and a hemorrhagic diarrhea case from Japan, respectively. However, low similarity was seen when comparing the Norwegian O145:H25 Stx2a phage to Stx2a phages from STEC of other serotypes. In all the Norwegian O145:H25 STEC, we identified a second phage or remnants of a phage (a shadow phage, 61 kbp) inserted at the same integration site as the Stx2a phage. The shadow phage shared similarity with the Stx2a phage, but lacked *stx2a* and harbored effector genes not present in the Stx2a phage. We identified a conserved Stx2a phage among the Norwegian O145:H25 STEC that shared integration site with a shadow phage in all isolates. Both phage and shadow phage harbored several virulence-associated genes that may contribute to the increased pathogenicity of O145:H25 STEC.

## Introduction

Infection with Shiga toxin-producing *Escherichia coli* (STEC) may lead to severe symptoms, such as hemorrhagic diarrhea and hemolytic uremic syndrome (HUS) (Karch et al., 2005). Approximately 5% of STEC infections in European Union (EU) countries and Norway lead to HUS (Brandal et al., 2015; Naseer et al., 2017; European Food Safety Authority European Centre for Disease Prevention Control, 2019; Jenssen et al., 2019). In the EU, STEC of serogroups O26, O157, O145, O80, and O111 are most commonly associated with HUS cases (European Food Safety Authority European Centre for Disease Prevention Control, 2019). A study investigating Norwegian STEC from 1992 to 2012 found that serogroups O157, O26, O145, and O103 were most frequently identified in HUS cases (Brandal et al., 2015). Within serogroup O145, serotype O145:H25, formerly described as O145:H?, is most commonly isolated from HUS cases in Norway (Brandal et al., 2015; Naseer et al., 2017; Jenssen et al., 2019).

Some subtypes of the Shiga toxin (Stx), the principal virulence factor of STEC, have a strong association with development of HUS, and subtype Stx2a is regarded as the most important of these (Brandal et al., 2015; De Rauw et al., 2018; Ylinen et al., 2020). In Norway, STEC with Stx subtypes Stx2a, Stx2c, and Stx2d are defined as high-virulent, and their identification triggers strict infection control measures including close follow-up of cases (Norwegian Institute of Public Health, 2021). The *stx* genes are encoded on bacteriophages (usually referred to as phages) integrated in the bacterial genome (prophage). Stx encoding phage (Stx phage) genomes are heterogeneous, varying in sizes from approximately 29 to 72 kbp, but prophages of over 100 kbp have been described (González-Escalona et al., 2019; Rodríguez-Rubio et al., 2021). The Stx2 phages have, however, a common modular arrangement of genes that can be divided into six functional parts: (1) recombination, (2) early regulation, (3) replication, (4) late regulation, (5) *stx2* and bacterial lysis, and (6) phage structural proteins (Plunkett et al., 1999; Recktenwald and Schmidt, 2002; Casjens, 2008). The *stx2* are, together with lysis genes, part of the late phage region, which also consists of genes related to prophage induction and phage replication (Tyler et al., 2004; Smith et al., 2012). The late phage region is controlled by several regulatory elements, such as the CI repressor, antiterminators Q and N, late phage promoter (pR’), and terminator (tR’), all encoded upstream of *stx2* (Tyler et al., 2004; Smith et al., 2012). The process leading to transcription of the late phage genes, including *stx2*, is induced by the onset of the bacterial SOS response. The bacterial SOS response can be activated by, for instance, exposure to Mitomycin C, UV irradiation, and antimicrobials (Kruger and Lucchesi, 2015). Antimicrobial treatment is therefore debated as treatment of STEC infections as the activated SOS response may lead to Stx phage induction and increased Stx production, aggravating the disease rather than treating it. The SOS response is activated by detection of single-stranded DNA (caused by DNA damage) that activates the RecA protein (Maslowska et al., 2019). RecA cleaves the CI repressor, which in brief leads to the expression of the antiterminators N and Q. Subsequently, Q binds to pR’, and transcription of the late phage genes starts (Pacheco and Sperandio, 2012). Variation in the phage genes, such as the pR’ region, *cI*, *q*, and other regulatory genetic elements, affects Stx2 expression and strain virulence (Tyler et al., 2004; Ogura et al., 2015; Olavesen et al., 2016; Zhang et al., 2018). The Stx phages may also contain genes for other virulence and/or fitness factors (Barondess and Beckwith, 1995; Smith et al., 2012). Thus, the variability in Stx phage characteristics indicates that the phages may influence the virulence potential of STEC across different bacterial hosts.

Despite originating from STEC of different serogroups, some Stx2a phages cluster closely together with respect to single nucleotide polymorphism (SNP) differences. However, other Stx2a phages originating from STEC of the same serogroup are clearly different, suggesting that Stx2a phages are not necessarily serogroup specific (Zhang et al., 2019). Stx2a phages from O145:H25 STEC isolated from HUS patients in the USA have been found to have a different genomic structure compared with the well characterized O157:H7 Sakai Stx2a phage (Lorenz et al., 2017).

The high occurrence of HUS in Norwegian patients with STEC O145:H25 infections is not well understood. We hypothesize that characteristics of the Stx2a phages found in the Norwegian O145:H25 strains might contribute to this phenomenon. The aim of this study was therefore to explore the pathogenic potential of the Stx2a phage genome extracted from whole genome sequences of Norwegian O145:H25 STEC and to compare these with previously published Stx2a phages of O145 and non-O145 STEC. We employed Oxford Nanopore Technology (ONT) long read sequencing, together with Illumina short read sequencing, and performed a detailed *in silico* characterization of Stx2a encoding phages from eight STEC O145:H25 isolated from patients with hemorrhagic diarrhea and HUS in Norway between 2007 and 2018.

## Materials and Methods

### NIPH O145:H25 STEC Isolates

**FIGURE 1:**
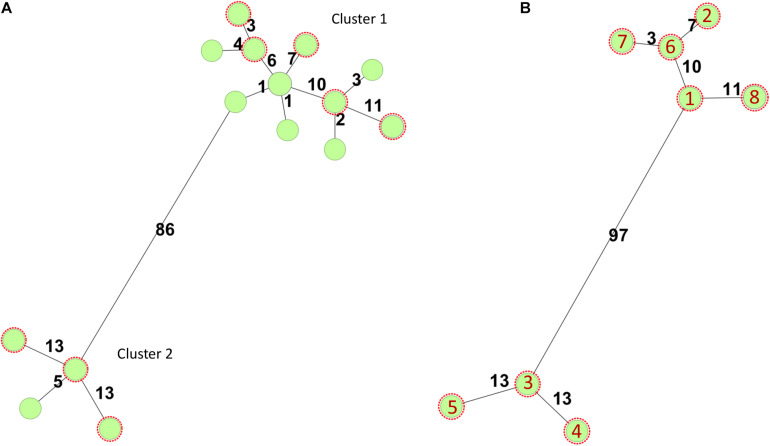
Minimum-spanning trees based on core genome multilocus sequence typing (cgMLST) using the *Escherichia*/*Shigella* scheme v.1 (2,513 targets) from Enterobase run in SeqSphere+7.0.6 (Ridom GmbH, Münster, Germany). Number of allelic differences is shown on the connecting lines between the different strains. All STEC were of sequence type 342. **(A)** Minimum-spanning tree of 18 *stx2a* positive O145:H25 STEC strains whole genome sequenced at the National Reference Laboratory between 2007 and 2020. Two of the circles include two or three isolates, respectively. The O145:H25 STEC fell into two clusters (86 allelic differences). Cluster 1 contained 14 STEC from 2009 to 2020 and cluster 2 four STEC from 2007, 2011, and 2012. Both clusters harbored isolates from HUS cases. **(B)** Minimum-spanning tree of O145:H25 STEC (*n* = 8) selected for in-depth analysis. Red numbers refer to the different isolates (with year of isolation in parentheses): 1 = NIPH-O145:H25-001 (2013), 2 = NIPH-O145:H25-002 (2009), 3 = NIPH-O145:H25-003 (2007), 4 = NIPH-O145:H25-004 (2011), 5 = NIPH-O145:H25-005 (2012), 6 = NIPH-O145:H25-006 (2014), 7 = NIPH-O145:H25-007 (2018), and 8 = NIPH-O145:H25-008 (2015).

In Norway, all presumptive human clinical STEC isolates are submitted by the medical microbiology laboratories to the Norwegian Reference Laboratory (NRL) for Enteropathogenic Bacteria at the Norwegian Institute of Public Health (NIPH) for verification and characterization. In total, 40 *stx2a*, *eae* positive O145:H25 STEC, have been received at the NRL from 2007 to 2019, 12 (30%) isolated from HUS cases. All cases with available travel history, except two, were domestically acquired. As part of the national surveillance program of STEC, 18 O145:H25 STEC have been whole genome sequenced, and they all have sequence type (ST) 342 and fall into one of two clusters defined by core genome multilocus sequence typing (cgMLST) (Figure 1). Both clusters contain isolates from HUS cases. For this study, eight clinical O145:H25 STEC were selected as representatives of both clusters and different years of isolation. The strains were designated NIPH1–NIPH8 and are presented in Table 1.

**TABLE 1:** Characteristics of Stx2a phages from Shiga toxin-producing *E. coli* (STEC) of different serotypes examined in this study.

		**STEC**		**Stx2a phage**				
**Strain**	**Serotype**	**Country**	**Isolation year**	**Size (bp)**	**Integration site**	***q*-gene variant**	**Accession no.**	**References**
NIPH1	O145:H25	Norway	2013	45,261	*yciD*	*qO111*	GCA_910593615	
NIPH2^a^	O145:H25	Norway	2009	∼45,000	*yciD*	*qO111*	GCA_910593925	
NIPH3^a^	O145:H25	Norway	2007	∼45,000	*yciD*	*qO111*	ERR6073320	
NIPH4	O145:H25	Norway	2011	∼45,000	*yciD*	*qO111*	ERR6073321	
NIPH5	O145:H25	Norway	2012	∼45,000	*yciD*	*qO111*	ERR6073322	
NIPH6	O145:H25	Norway	2014	∼45,000	*yciD*	*qO111*	ERR6073323	
NIPH7	O145:H25	Norway	2018	∼45,000	*yciD*	*qO111*	ERR6073324	
NIPH8	O145:H25	Norway	2015	∼45,000	*yciD*	*qO111*	ERR6073325	
12E129	O145:H28	Japan	na^b^	45,509	na	*qO111* ^c^	LC567841	
CFSAN004176	O145:H25	United States	2003	44,992	*yecE*	*qO111*	NZ_CP014583^*d*^	Lorenz et al., 2017
EDL933	O157:H7	United States	1982	61,648	*wrbA*	*q933*	AF125520	Plunkett et al., 1999
Sakai	O157:H7	Japan	1996	60,942	*wrbA*	*q933*	NC_000902	Makino et al., 1999
German outbreak strain	O104:H4	Germany	2011	60,894	*wrbA*	*q933*	NC_018846	Beutin et al., 2012
Norwegian outbreak strain	O103:H25	Norway	2006	60,523	*wrbA*	*q933*	NC_019442	L’Abee-Lund et al., 2012
O26:H11	O26:H11	na	na	62,207	na	*q933* ^*c*^	AB609719	

*^*a*^Strains NIPH2 and NIPH3 have also been sequenced previously and published under the accession numbers ERS480183 and ERS480155, respectively (Haugum et al., 2014).*

*^*b*^na = not applicable.*

*^*c*^Based on the alignment made in this study.*

*^*d*^Accession number of the strain.*

### Whole Genome Sequencing

The STEC strains included (NIPH1–8) were previously sequenced with the Illumina platform (MiSeq or NextSeq; Illumina, Inc., San Diego, CA, United States) at the NIPH for surveillance purposes. The Illumina sequences for NIPH1–NIPH8 can be found under the accession numbers: ERR6073319, ERR6093688, ERR6073320, ERR6073321, ERR6073322, ERR6073323, ERR6073324 and ERR6073325, respectively.

In addition to Illumina-based short reads, long read sequencing was conducted to gain large enough contigs for extraction of complete phages. Therefore, STEC NIPH1 and NIPH2 were also sequenced using Oxford NanoPore Technologies (Oxford, United Kingdom).

DNA for long reads sequencing was isolated using Wizard^®^ Genomic DNA Purification Kit (Promega, Madison, WI, United States) according to the manufacturer’s instructions. Sequencing was conducted on Oxford NanoPore Technologies MinION MK1B (Oxford, United Kingdom) sequencer using SQK-RBK004 Rapid Barcoding Kit and MinION SpotON R9.4.1 flow cell. Live basecalling was conducted using the MinKNOW (19.10.1) software (72 h). The run was according to the Oxford Nanopore Technologies standard protocol.

### Assembly and Annotation

Illumina raw reads adapters were removed by Trimmomatic (v.0.39) default mode (Bolger et al., 2014). FLASH (v.1.2.11) was used for generation of longer contigs, with max overlap set to 300 (Magoč and Salzberg, 2011). SPAdes (v.3.15.0) was used for main assembly with careful mode (Prjibelski et al., 2020). Quast 5.1 was used for quality control of SPAdes assembly (Gurevich et al., 2013).

Porechop (v.0.2.4, Ryan Wick^1^) was used for demultiplexing and to remove adapters from long reads. Hybrid assembly of long and short reads of NIPH1 and NIPH2 was conducted using Unicycler (v.0.4.8) (Wick et al., 2017). Canu (v.2.1) was used for long reads *de novo* assembly of NIPH2 only (Koren et al., 2017).

The hybrid assembled sequences for NIPH1 and NIPH2 can be found under the accession numbers GCA_910593615 and GCA_910593925, respectively. The Canu assembly of NIPH2 can be found under the accession number GCA_910593955.

### Identification and Characterization of Stx2a Phages From NIPH O145:H25 STEC

Annotation of the strains and phages was done using Prokka (v.1.14.5) and supplemented with NCBI blastx results for ORF/CDS with no assigned function (Seemann, 2014).

Extraction of the Stx2a phage from NIPH1 was conducted on the hybrid assembly of NIPH1, and the extracted Stx2a phage was used as a reference for extraction of the phage sequences from the remaining O145:H25 STEC isolates. The position of *stx2a* was located, and the area around was investigated for phage-related genes. Identification of the integration site was performed by locating the gene adjacent to the phage integrase gene and comparing this to other previously identified integration sites. In addition, the integration sites were BLASTed against the phage area to identify the site on both ends (Altschul et al., 1990). The Stx2a phage was assumed to be encoded within the insertion site, and the contents of the phage were compared and controlled against an already described O145:H25 Stx2a phage (Lorenz et al., 2017). NIPH2 genome was BLASTed against the identified NIPH1-Stx2a-phage to identify a similar phage sequence and integration site. For NIPH3–8 only Illumina sequences were available, and the Stx2a phages were extracted by reference-based assembly using the NIPH1-Stx2a-phage as reference. Reference-based assembly was conducted using Bowtie2 and SAMtools (v.1.11) (Li et al., 2009; Langmead and Salzberg, 2012). Seqtk (v.1.3^2^) was used to convert files from SAMtools’ mpileup to fasta. The reference assemblies were controlled for non-detected bases (n). Long contigs at the end of the predicted Stx2a phages also made it possible to identify insertion sites for Stx2a phages of STEC with only short read sequences (NIPH3–8).

Identification of *q*-gene type was conducted with BLAST against known *q*-genes (*qO111*, *q933*, *q21*) (Unkmeir and Schmidt, 2000; Lejeune et al., 2004; Haugum et al., 2012; Olavesen et al., 2016). Copy number variants (CNVs) of strains, phages, and *stx2a* were found using the SAMtools depth function (v.1.11), and the results were analyzed using R (v.3.6.3, R Core Team, 2020) (Li et al., 2009).

### Comparison of Stx2a Phages From NIPH O145:H25 STEC

The NIPH1–NIPH8-Stx2a phage sequences were compared phylogenetically. SNPs were extracted from the phage sequences using ParSNP and aligned using MAFFT (v.7.475) in auto mode (Katoh and Standley, 2013; Treangen et al., 2014). The output was used to make maximum likelihood tree with IQ-TREE (v.1.6.12) (Nguyen et al., 2014) with ultrafast bootstrapping approximation (UFBoot) (Hoang et al., 2017) and visualized by FigTree (v.1.4.4) (Andrew Rambaut, Institute of Evolutionary Biology, University of Edinburgh, Edinburgh^3^). The Stx2a phage sequences were also compared using BLAST Ring Image Generator (BRIG) alignment with default settings (Alikhan et al., 2011).

### Comparison of Stx2a Phages From NIPH O145:H25 STEC With Publicly Available Stx2a Phages From STEC of HUS Associated Serotypes

For comparison, Stx2a phages originating from STEC strains of five different serogroups O157, O145, O103, O26, and O104 were downloaded from the NCBI Nucleotide database. These particular Stx2a phages were selected according to the following reasons: O157:H7 and O26:H11 STEC are most frequently associated with HUS worldwide, O145 is among the top five STEC serogroups seen in HUS cases in Europe (especially O145:H28), O104:H4 STEC caused a large HUS outbreak in Europe in 2011, and O103:H25 STEC was responsible for a large HUS outbreak in Norway in 2006 (Schimmer et al., 2008; Frank et al., 2011; European Food Safety Authority European Centre for Disease Prevention Control, 2019). Details and accession numbers are shown in Table 1.

The Stx2a phage of NIPH1 was BLASTed against the NCBI Nucleotide Bacterial Viruses database (taxid:28883), and the best match in terms of highest query cover in combination with high identity was selected for comparison. Additionally, a Stx2a phage from an American O145:H25 STEC isolated from a HUS case previously published (NZ_CP014583.1) was included for comparison (Lorenz et al., 2017). The phage was extracted from the genome using the same approach as for the NIPH prophages described above.

### Visualization Tools

EasyFig (v3.14) Sullivan et al. (2011) was used for visualization of the phage genomes and for blastn comparison of genomic similarities between the different phages based on the GenBank gbk-files retrieved from Prokka or downloaded from NCBI.

## Results and Discussion

### Phylogeny of the NIPH O145:H25 Stx2a Phages

The genome sequences of eight high-virulent O145:H25 Stx2a producing *E. coli* strains were used for extraction of their Stx2a phage genomes and for analysis and comparison of their sequences.

The Stx2a phages extracted from O145:H25 STEC NIPH1–NIPH8 were closely related, with a size of approximately 45 kbp and with approximately 200 SNPs difference (Supplementary Figure 1). Based on topology of the SNP phylogeny, the Stx2a phages appeared to form two separate cluster groups, but this was not supported by the bootstrap values. Moreover, the BRIG alignment showed that these phages were close to identical (Supplementary Figure 2). However, the assembly of NIPH3–8-Stx2a-phages was all based on NIPH1-Stx2a-phage as reference, and thus only genomic regions present in the reference genome were compared. Furthermore, the assembled Stx2a phages may consist of sequences originating from other phages than the one of interest inserted in the bacterial genome, resembling the Stx2a phage used as reference. NIPH2-Stx2a-phage was not reference assembled but extracted from the hybrid assembly using NIPH1-Stx2a-phage as template and is discussed below.

### Genomic Structure and Characterization of NIPH-Stx2a-Phages

**FIGURE 2:**
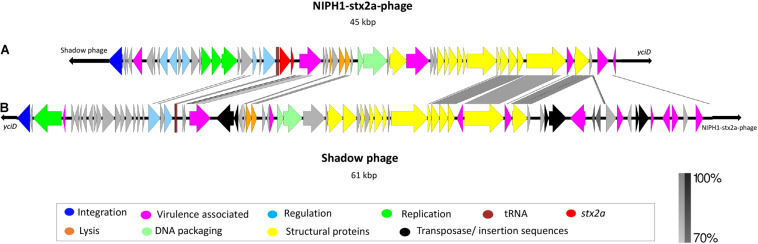
Description and alignment of the two identified phages at integration site *yciD* in NIPH1 O145:H25 STEC. **(A)** The Stx2a phage (NIPH1-Stx2a-phage), size 45 kbp. **(B)** Remnants of a phage with the same genomic organization as the NIPH1-Stx2a-phage, but without the *stx2a* gene, referred to as shadow phage. It is 61 kbp long. The shadow phage is placed upstream of the NIPH1-Stx2a-phage in the integration site *yciD*. Several of the genes encoding structural proteins, tRNAs, and lysis genes show higher than 70% homology at nucleotide level between the two phages. However, the shadow phage harbors several different effector genes not present in the NIPH1-Stx2a-phage.

The modular structure of the NIPH-Stx2a-phage genomes was comparable with previously published Stx2a phages, and the genes were divided into six modules: integration, early regulation, replication, late regulation upstream of *stx2a*, and lysis followed by genes encoding structural proteins (Figure 2). The *q*-gene variant of the NIPH-Stx2a-phages was *qO111*, a variant that has previously been associated with Norwegian high-virulent sorbitol fermenting O157:H7 STEC (Haugum et al., 2014). The NIPH-Stx2a-phages were inserted in the *yciD* gene, a 639 bp long putative outer membrane protein found adjacent to the phage integrase gene. The *yciD* gene was identified on both sides of the Stx2a phage, as a complete 639 bp gene on the 5’ side and a 119 bp sequence on the 3’ side. Interestingly, what appears to be another phage, was also inserted within the integration site of all NIPH O145:H25 strains analyzed (discussed below). Many Stx phage integration sites have been described in STEC carrying the locus of enterocyte effacement (LEE) (Ogura et al., 2007; Steyert et al., 2012). Despite the fact that *yciD* previously only has been identified as integration site in LEE-negative STEC, we detected *yciD* as integration site for Stx2a phages in LEE-positive STEC (Steyert et al., 2012). In our study, STEC NIPH1–NIPH8 were also the only STEC using *yciD* as integration site for the Stx2a phages (Table 1). These findings suggest that the Stx2a phages of the Norwegian high-virulent O145:H25 STEC are most likely of the same phage type and they were present in O145:H25 STEC from both cgMLST clusters (Figure 1), indicating that this Stx2a phage variant likely is dominating in Norwegian human clinical O145:H25 STEC isolates.

All O145:H25 STEC included in the study contained one Stx phage and one copy of *stx2a* according to the CNV analysis (Supplementary Table 1). Previous studies have shown that Norwegian O145:H25 STEC from human clinical cases carry *stx2a* and *eae*, encoding the adhesion factor intimin (Brandal et al., 2015; Jenssen et al., 2019). Interestingly, data from NIPH have revealed that O145:H25 *E. coli* carrying *eae* without *stx2a* have been isolated from HUS cases. These *eae*+ *E. coli* isolates cluster together with O145:H25 STEC based on cgMLST, indicating that the Stx2a encoding phage might have been lost (Bielaszewska et al., 2008; Ferdous et al., 2015; Senthakumaran et al., 2018).

The NIPH-Stx2a-phages also contain other genes, some associated with virulence properties, such as serine protease, 9-O-acetyl-N-acetylneuraminic acid deacetylase, Clp protease, non-LEE encoded effector C (NleC), and damage inducible protein I (DinI) (Supplementary Table 2). 9-O-acetyl-N-acetylneuraminic acid deacetylase has previously been associated with the ability to grow in the presence of sialic acid, commonly found in mammalian mucosal sites (Steenbergen et al., 2009). NleC is a non-LEE encoded effector protein or a type III secretion protease. When injected into the host by the type III secretion system, NleC will inhibit the activation of the innate immune response (Li et al., 2014). DinI is activated by DNA damage and will inhibit RecA, suppressing the SOS response (Yasuda et al., 2001). If the predicted *dinI* is expressed, it will ultimately limit the induction of the Stx2a phage if the bacterial SOS response is activated.

Serine protease and Clp protease are known to degrade proteins; however, it is not clear whether these proteases have a part in the phage biosynthesis or have a virulence-associated function (Dokland, 1999; Wegrzyn et al., 2000). Further studies are needed to elucidate whether these genes are expressed or not, but when expressed, they will benefit the bacterial host during human epithelial infection, possibly increasing the severity of the disease.

### Comparison of the Genomic Structure of NIPH1-Stx2a-Phage With Stx2a Phages From Previously Published O145 STEC

We compared the extracted NIPH1-Stx2a-phage genome with two other O145 Stx2a phage genomes; one identified by the BLAST search showing the highest sequence coverage and sequence homology and the other identified through a previous publication (Lorenz et al., 2017).

**FIGURE 3:**
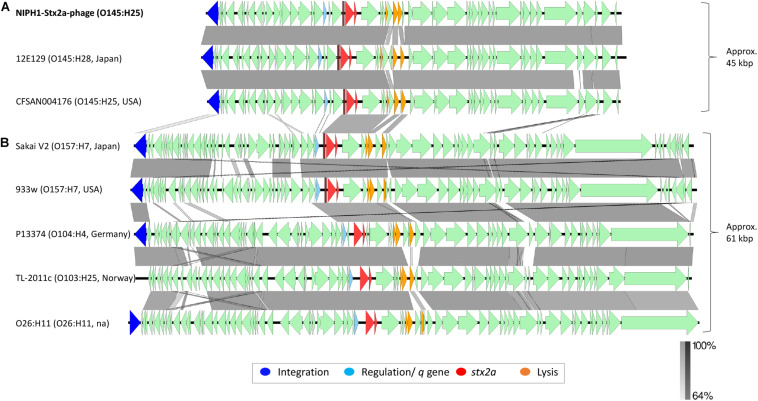
Comparison of the genome structure of the O145:H25 NIPH1-Stx2a-phage with Stx2a phages from STEC of various serotypes. The alignment shows separation of the Stx2a phages in two groups, **(A)** one of approximately 45 kbp size and **(B)** the other of 61 kbp size. The genomic organization of genes is similar, with gene for integration followed by regulation of tRNAs, *stx2a*, lysis, and structural genes. There are large differences at the gene level between the two Stx2a phage groups and smaller differences within the groups. Left scale bar shows percent sequence homology.

The NCBI BLAST search showed high homology (query coverage 98.0%, identity 98.19%) between the NIPH1-Stx2a-phage and an O145:H28 Stx2a phage isolated from STEC case of hemorrhagic diarrhea in Japan (NCBI accession no. LC567841.1) (Nakamura et al., 2020). The NIPH1-Stx2a-phage also exhibited high homology (query coverage 94.0%, identity 99.64%) with a Stx2a phage extracted from a previously published O145:H25 STEC (CFSAN004176, accession no. NZ_CP014583) from a HUS case in the USA (Lorenz et al., 2017). Similar to the NIPH1-Stx2a-phage, both these Stx2a phages were approximately 45 kbp long and shared integration site with remnants of another phage that we describe below. The alignment of these three O145 Stx2a phages is shown in Figure 3, as the top three alignments. Despite originating from different geographical locations (Norway, USA, and Japan), all three O145 Stx2a phages had high sequence similarity, with 94.0–97.0% query coverage and 98.16–99.63% identity. They all encode the same integration gene, *q*-gene and almost all other genes. The most notable difference between the Japanese O145:H28 Stx2a phage and the other two O145:H25 Stx2a phages was that the Japanese phage had two hypothetical genes upstream of the two last lysis genes, whereas the American O145:H25 Stx2a phage and NIPH1-Stx2a-phage both had one hypothetical gene between the two last lysis genes. The Japanese and the American Stx2a phages also differed on sequence level of the *lom* (lambda outer membrane) gene. The high sequence homology between these O145 Stx2a phages indicates that the 45 kbp Stx2a phage group was conserved across O145 STEC isolates from clinical STEC associated with bloody diarrhea and HUS.

However, Stx2a phages similar to the EDL933 non-sorbitol fermenting (NSF) O157:H7 Stx2a phage have also been identified in O145 STEC, indicating that O145 STEC may carry a Stx2a phage similar to NIPH1-Stx2a-phage or similar to 933W Stx2a phage (Lorenz et al., 2017; Krüger et al., 2018; Carter et al., 2021). Isolates with O145:H28 Stx2a phage similar to NIPH1-Stx2a-phage are not shown to have higher production of Stx2a compared with O145:H28 STEC with Stx2a region similar to the one found in the O157:H7 EDL933 phage when induced by, for example, enrofloxacin (Carter et al., 2021).

### Comparison of the Genomic Structure of NIPH1-Stx2a-Phage With Stx2a Phages From STEC of HUS Associated Serotypes

We compared the NIPH1-Stx2a-phage with Stx2a phages from STEC of different serotypes associated with HUS by aligning nucleotide sequences (Figure 3). This alignment showed a clear division of Stx2a phages in two groups, one of 45 kbp size, including the O145 Stx2a phages, and another of 61 kbp size containing Stx2a phages from NSF O157:H7, O26:H11, O104:H4, and O103:H25 STEC. The two Stx2a phage groups were considerably heterogeneous with respect to gene content (most genes with less than 64.0% sequence similarity at nucleotide level). The two different groups of Stx2a phages exhibited similarities of >64.0% in the *q*-genes and integrase genes; however, these genes were not of the same variant. The 61 kbp Stx2a phages carried antiterminator gene *q933*, whereas the 45 kbp Stx2a phages carried *qO111* (Table 1 and Figure 3). The 61 kbp phages with known integration site were all incorporated into *wrbA*, while the 45 kbp phages used *yciD* or *yecE* (Plunkett et al., 1999; Beutin et al., 2012; L’Abee-Lund et al., 2012; Lorenz et al., 2017). There were also similarities between the tRNAs, *stx2a* genes, lysis genes, and some hypothetical genes located between the *stx2a* genes and the lysis genes. The *stx2a* genes were identical in NIPH1-Stx2a-phage and O104:H4 Stx2a phage, and these differed with only 1 bp to the *stx2a* in other 61 kbp Stx2a phages (pos. 867, C in NIPH and T in the 61 kbp group). The NIPH1-Stx2a-phage *stx2a* gene differed in 10 bp from the other two O145 Stx2a phages that were identical to each other. The remaining genes of the phages did not have any similarity above 64.0% nucleotide identity. Between the *q*-gene and the tRNAs, the 45 kbp phage group had a transcriptional regulator and site-specific DNA methylase (regulates/modulates DNA), which were not present in the 61 kbp group of Stx2a phages; a similar observation has been described by Carter et al. (2021).

Differences in gene content were also detected within the 61 kbp Stx2a phage group in concordance with previous observations (Smith et al., 2012; Ogura et al., 2015; Yin et al., 2015; Llarena et al., 2021). These differences, however, were found to be minor compared with the differences seen between the 45 kbp and the 61 kbp Stx2a phage groups described in our study (Figure 3).

### The Shadow Phage of NIPH1

The NIPH1 shadow phage was located upstream of the integrase gene of the NIPH1-Stx2a-phage. The size of the shadow phage genome was approximately 61 kbp. The shadow phage had similar modular structure as Stx2 phages, but without *stx* genes or remnants of these (Figure 2). The shadow phage also contained Stx phage-associated elements, such as *lom* protein, two genes annotated as antiterminator Q (but none of them matched the previously described *q*-gene variants), tRNAs, integrase gene, and several IS elements. The NIPH1 shadow phage contained a high amount of different non-LEE encoded effectors not seen in the Stx2a phage, *nleG*, *nleF*, *nleA*, and the *E. coli* secreted effectors *espO* and *espM* (Supplementary Table 2). All these effectors have the potential ability to increase pathogenicity by inhibiting, degrading, or impacting the host cells’ critical functions by, for example, targeting important proteins for cell function or defense mechanisms against infecting pathogens (Kim et al., 2007; Arbeloa et al., 2009; Blasche et al., 2013; Berger et al., 2018; Valleau et al., 2018). The effector genes in the shadow phage are all close to transposons or IS elements, suggesting the possibility of horizontal gene transfer of these genes.

Similarity between some of the phage structural genes with more than 70% identity at the nucleotide level was seen between the Stx2a phage and the shadow phage of NIPH1, but the *q*-genes and integrase gene had less than 70% identity (Figure 2). However, both phages had similarity in the *nanS* gene (9-O-acetyl-N-acetylneuraminic acid deacetylase) and the *lom* gene. Many phages contain similar and repeated regions, and similarity found in the structural proteins and the *q*-genes between NIPH-Stx2a-phage and shadow phage might suggest that the latter also is a lamda phage.

NCBI BLAST search with the shadow phage from NIPH1 detected a match with another probable shadow phage present in the O145:H28 STEC from Japan (accession no. LC567841.1) described above. The two shadow phages had 97.7% sequence identity. The Stx2a phages of the two HUS O145:H25 STEC described by Lorenz et al. (2017) also shared integration site with what appears to be the remnants of another phage. However, this phage looked quite different to our shadow phage according to the annotation, and it contained remnants of the *stx2a* gene, in contrast to our shadow phage (Lorenz et al., 2017). Shared integration site between the Stx2a phage and remnants of or another (shadow) phage appeared to be a common phenomenon in O145 STEC from humans with severe disease. Whether the shadow phages are active or may be induced is currently not clear as several parts found in the Stx2a phages were missing from these. Thus, further analyses are needed.

### NIPH2-Stx2a-Phage of NIPH2, an Exception

The *de novo*, long reads only, assembly managed to completely close the NIPH2 STEC genome. Alignment using the NIPH1-Stx2a-phage as a reference showed that the NIPH2-Stx2a-phage was present, but divided in two and located at different locations in the genome. As previously mentioned, this Stx2a phage was almost identical to the NIPH1-Stx2a-phage. The two halves were separated by approximately 500 kbp, in the middle of what was probably the gene encoding the terminase small subunit. The first part of the NIPH2-Stx2a-phage (size 21.7 kbp) included the integrase gene and the *stx2a* gene, whereas the second part (size 23.5 kbp) contained the integration site *yciD* and structural genes, together with a shadow phage, almost identical to the shadow phage present in the NIPH1 strain. We know from previous studies that NIPH2 did produce Stx and lysed, but if, or how, this affects the creation and assembly of the Stx2a phage body of this strain is not known (Ramstad et al., 2020). The organization of this Stx2a phage within the STEC genome might be due to the frequent recombination observed in *E. coli* (Fitzgerald et al., 2020). A previous study investigating prophage-dependent recombination in O157:H7 *E. coli* found large chromosomal rearrangements by inversion, and most were bounded by prophages. When aligning the contig of NIPH1 which includes the Stx2a phage with the closed genome of NIPH2, we found a large inversed part, in the middle of the Stx2a phage of NIPH2. This shows that this phenomenon also occurs in O145:H25 STEC (Supplementary Figure 3; Fitzgerald et al., 2020). Further investigation to see if the bacteria produced a complete Stx2 phage when induced should be conducted.

## Conclusion

This study was based on the hypothesis that characteristics of the Stx2a phages might explain the high occurrence of HUS in Norwegian patients with STEC O145:H25 infections. To support or eliminate this hypothesis, we sequenced the genomes of eight Norwegian high-virulent O145:H25 STEC, all encoding Stx2a and isolated from clinical cases with bloody diarrhea or HUS. From these sequences, we extracted the putative Stx2a phage genomes for closer investigation. The high sequence homology found between the Stx2a phages indicates that one Stx2a phage type dominates among the human clinical O145:H25 STEC pool. This O145:H25 Stx2a phage also shared high homology with other published Stx2a phages from O145:H28 and O145:H25 STEC isolated from other countries, illustrating that this phage is not unique to Norwegian STEC. The O145 Stx2a phages were approximately 45 kbp in size and formed a distinct group with low sequence homology to a group of larger (>60 kbp) Stx2a phages from STEC of other serotypes associated with HUS.

An interesting finding was that the Stx2a phage of the Norwegian O145:H25 STEC shared integration site with the remnants of another phage (a shadow phage without *stx*) with somewhat similar structural proteins. Further analysis revealed a similar phenomenon in O145:H28 and O145:H25 STEC from Japan and USA, respectively. Both the shadow phage and the Stx2a phage carried a spectrum of different virulence genes. Supportive of our hypothesis, we speculate whether interaction between the two phages could contribute to the increased pathogenicity observed for Norwegian O145:H25 STEC. Characteristics of the Stx2a phages might explain the high occurrence of HUS in Norwegian patients with STEC O145:H25 infections, but more research is needed.

## Data Availability Statement

The original contributions presented in the study are publicly available. This data can be found here: https://www.ebi.ac.uk/ena under the following accession numbers: ERR6073319, ERR6073320, ERR6073321, ERR6073322, ERR6073323, ERR6073324, ERR6073325, ERR6093688, GCA_910593615, GCA_910593925 and GCA_910593955

## Author Contributions

SR participated in planning and designing of the study, performed the *in silico* work and was involved in the interpretation of the results, and reviewed the literature and wrote the first draft of the manuscript and revision. AT, JBj, and B-AL participated in planning and designing of the conducted research, interpreted the data, and reviewed and wrote the manuscript and approved the final version. JBo participated in planning and designing of the study, performed the *in silico* work and was involved in the interpretation of the results, and reviewed and wrote the manuscript and approved the final version. YW supervised in planning of the study and in the analyses of the obtained results and supervised during the writing/revision of the manuscript and approved the final version. LB had the idea of the project, designed the project together with the other authors, selected the material, participated in the interpretation of the results, and reviewed and wrote the manuscript and approved the final version. All authors approved the submitted version of the article.

## Conflict of Interest

The authors declare that the research was conducted in the absence of any commercial or financial relationships that could be construed as a potential conflict of interest.

## Publisher’s Note

All claims expressed in this article are solely those of the authors and do not necessarily represent those of their affiliated organizations, or those of the publisher, the editors and the reviewers. Any product that may be evaluated in this article, or claim that may be made by its manufacturer, is not guaranteed or endorsed by the publisher.

## Footnotes

1
https://github.com/rrwick/Porechop

2
https://github.com/lh3/seqtk

3
http://tree.bio.ed.ac.uk/software/figtree/
