# Supplementary material for: Characterization of Shiga Toxin 2a Encoding Bacteriophages Isolated From High-Virulent O145:H25 Shiga Toxin-Producing Escherichia coli
Source: Front Microbiol. 2021 Sep 8;12:728116. doi: 10.3389/fmicb.2021.728116 (PMC8456039; doi:10.3389/fmicb.2021.728116)
Supplement: Supplementary file 1 [file Presentation_1.pdf]

Supplementary Table 1. Copy number variants (CNV) of the Stx2a phage, ghost phage and *stx2a* gene in each of the eight Norwegian STEC O145:H25 strains.

	CNV			
Strain name	Strain	Stx2a phage	Ghost phage	*stx2a* gene
NIPH-O145:H25-001	49.11608	195.99	248.4843	33.7837
NIPH-O145:H25-002	34.96262	119.0602	148.0148	39.3547
NIPH-O145:H25-003	149.06071	381.376	522.48	115.74
NIPH-O145:H25-004	121.27959	594.628	732.161	66.679
NIPH-O145:H25-005	41.5722	201.8236	212.152	67.399
NIPH-O145:H25-006	135.23445	620.131	762.471	75.7725
NIPH-O145:H25-007	88.61372	415.663	543.225	48.7342
NIPH-O145:H25-008	137.5677	698.06	811.75	79.0918

Supplementary Table 2. Proteins identified in NIPH-Stx2a-phage and shadow phage by either Prokka or NCBI BLAST.

**NIPH-Stx2a-phage**	**Shadow phage**
Integrase IntQ	Integrase
Hypothetical protein	Hypothetical protein
Hypothetical protein	Exodeoxyribonuclase
Hypothetical protein	Killing protein KilR
Serine protease	Hypothetical protein
Hypothetical protein	Hypothetical protein
Hypothetical protein	Hypothetical protein
Hypothetical protein	Hypothetical protein
Transcriptional regulator	Hypothetical protein
Phage repressor protein CI	Hypothetical protein
Hypothetical protein	Hypothetical protein
DNA binding protein or putative antirepressor	Hypothetical protein
Hypothetical protein	Hypothetical protein
Hypothetical protein	Hypothetical protein
Phage replication protein O	Hypothetical protein
DNA helicase	Hypothetical protein
Replicative DNA helicase	Hypothetical protein
Hypothetical protein	Hypothetical protein
Hypothetical protein	Antiterminator protein Q
Antiterminator Q	Hypothetical protein
Hypothetical protein	Antiterminator Q
DNA methylase	tRNA-Met
tRNA-Met	tRNA-Lys
tRNA-Arg	tRNA-Arg
tRNA-Arg	Hypothetical protein
Shiga toxin subunit A	Hypothetical protein
Shiga toxin subunit B	9-O-acetyl-N-acetylneuraminic acid deacetylase
9-O-acetyl-N-acetylneuraminic acid deacetylase	IS66 family transposase ISEc8
Hypothetical protein	TnpB IS66 family insertion sequence element
Hypothetical protein	Hypothetical protein
Putative phage holin protein	Hypothetical protein
Hypothetical protein	Lysozyme RrrD
Lysozyme RrrD or endolysin	Endopeptidase
Lysis protein or endopeptidase	Hypothetical protein
Hypothetical protein	Hypothetical protein
Terminase small subunit	Dnase
Terminase large subunit	Phage terminase small subunit
Hypothetical protein	Phage terminase large subunit
Phage portal protein	Hypothetical protein

ATP-dependent Clp protease proteolytic subunit	Hypothetical protein
Hypothetical protein	Portal protein
Hypothetical protein	Portal protein
Minor tail protein Z	Hypothetical protein
Minor tail protein U	Head-tail adaptor
Tail assembly protein	Hypothetical protein
Minor tail protein	Minor tail protein
Minor protein	Minor tail protein
Tail length tape measure protein	Tail assembly chaperone
Minor tail protein	Hypothetical protein
Minor tail protein	Tail tape measure protein
Tail assembly protein	Tail protein
Tail assembly protein	Minor tail protein
Phage host specificity protein	Phage tail assembly protein
Outer membrane protein X/ Lom	Phage tail assembly protein
Tail fiber protein	Superoxide dismutase [Cu-Zn] 1
Hypothetical protein	Phage host specificity protein
NleC	Attachment invasion locus protein Lom
DNA damage-inducible protein I	Tail fiber protein
	Hypothetical protein
	Hypothetical protein
	TnpB IS66 family insertion sequence accesory prot
	IS66 family transposase ISEc8
	NleA
	Hypothetical protein
	Hypothetical protein
	Hypothetical protein
	Effector protein NleF
	Hypothetical protein
	IS3 family transposase ISEc17
	IS3 family transposase ISEc17
	EspO
	NleG
	EspM
	Hypothetical protein
	NleG

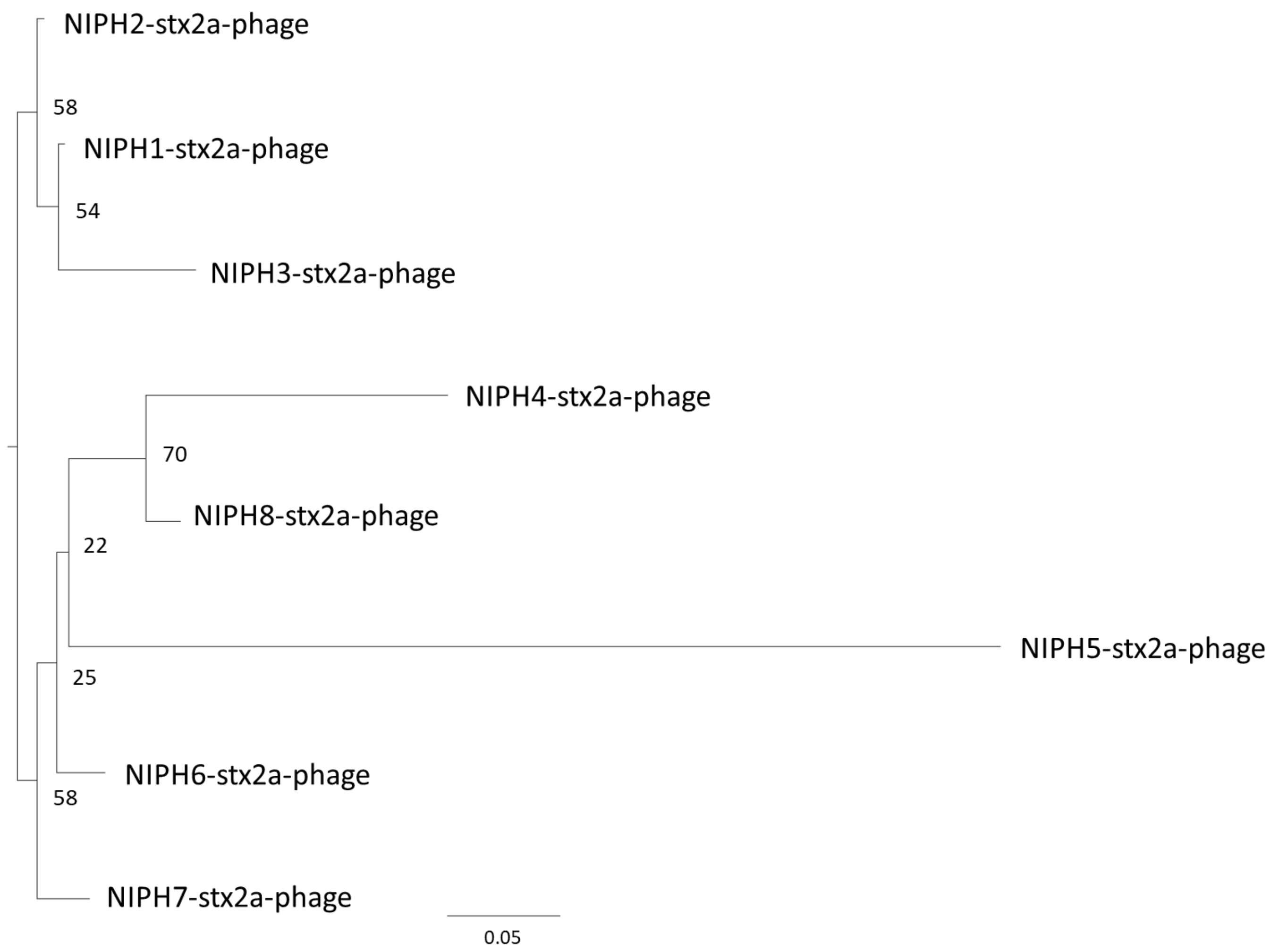

Supplementary Figure 1. Maximum likelihood tree of the Stx2a phages of Norwegian O145:H25 STEC (NIPH1-NIPH8) with approximately 200 single nucleotide polymorphisms (SNPs) difference. Shown are MAFFT alignment with ultrafast bootstrap (UFBoot) values generated by IQ-TREE. Values less than 90 are usually not considered significantly different by the UFBoot procedure.

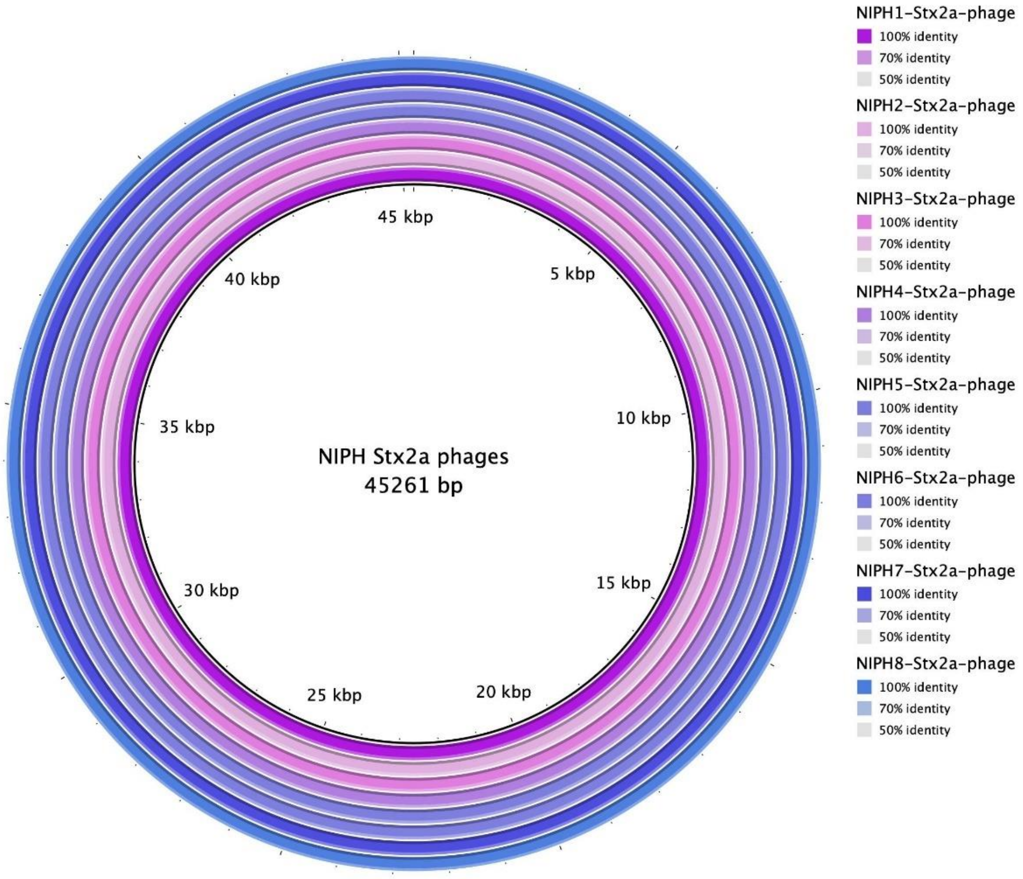

Supplementary Figure 2. BLAST Ring Image Generator, BRIG, alignment of the Stx2a phages of Norwegian O145:H25 STEC (NIPH1-NIPH8). The alignment shows high similarity between the Stx2a phages. NIPH1-Stx2a-phage was used as the reference genome, both for the phage assembly and the BRIG alignment.

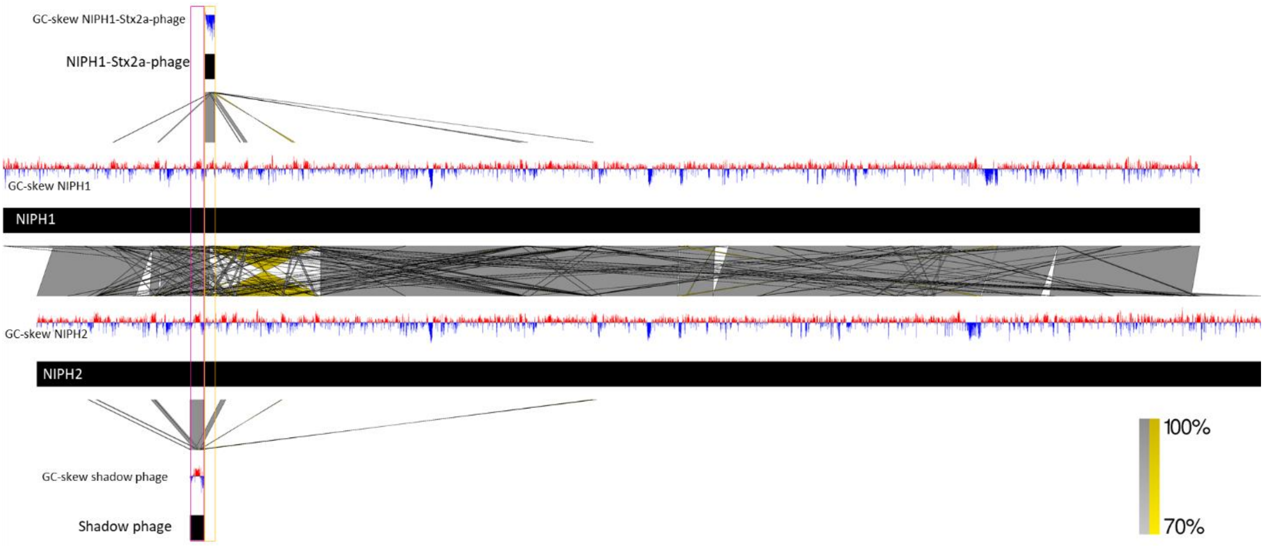

Supplementary Figure 3. EasyFig pairwise alignment of the largest contig of NIPH1 (5.4 mbp) with the chromosome of NIPH2 (5.5 mbp) together with NIPH1-Stx2a-phage and the shadow phage alignments, respectively. The genome sequences are shown as thick black vertical lines and above each sequence is the GC-skew shown in red for positive skew (G's abundant on the leading strand) and blue for negative skew (C abundance on the lagging strand). The pink box represents the location of the shadow phage in NIPH1/NIPH2 genome sequences while the yellow box represents the NIPH-Stx2a-phage. The bottom right legend indicates blast hit% (grey) and reverse complemented BLAST hit% (yellow). The inversion (colored yellow) occurring in NIPH2, with respect to NIPH1, splits the Stx2a phage in the middle into two separate parts with approximately 500 kb between them (see text for more details).
